# Supplementary material for: Single-molecule imaging correlates decreasing nuclear volume with increasing TF-chromatin associations during zebrafish development
Source: Nat Commun. 2018 Dec 6;9:5218. doi: 10.1038/s41467-018-07731-8 (PMC6283880; doi:10.1038/s41467-018-07731-8)
Supplement: Supplementary file 1 — Supplementary Information [file 41467_2018_7731_MOESM1_ESM.pdf]

# Supplementary Information

## Single-molecule imaging correlates decreasing nuclear volume with increasing TF-chromatin associations during zebrafish development

Matthias Reisser[1], Anja Palmer[1], Achim P. Popp[1], Christopher Jahn[2], Gilbert Weidinger[2] and J. Christof M. Gebhardt[1,*]

[1] Institute of Biophysics, Ulm University, Albert-Einstein-Allee 11, 89081 Ulm, Germany
[2] Institute of Biochemistry and Molecular Biology, Ulm University, Albert-Einstein-Allee 11, 89081 Ulm, Germany
[*]Corresponding author christof.gebhardt@uni-ulm.de

# Contents

**1 Supplementary Figures** **2**

**2 Supplementary Tables** **12**

**3 Supplementary Methods** **14**
Interlaced Time-Lapse Microscopy (ITM) . . . 14
3.1 Optimized temporal illumination scheme and classification of molecules . . 14
3.2 Linking the ratio stable to all bound molecules to quantitative values . . . . 14
3.2.1 Binding time distribution of fluorescent molecules in ITM . . . . . . . 14
3.2.2 Different starting points of the trajectories . . . 16
3.3 Correction for the percentage of all bound molecules . . . 17

**4 Supplementary Notes** **18**
Physical chemistry of chromatin-TF complex formation in the nucleus . . . . . . 18
4.1 Mathematical description of TF-DNA complex formation . . . 18
4.2 Exact solution for TF binding to two types of binding sites . . . 18
4.3 The bound fraction of TFs . . . 19
4.4 The bound fraction depends on the nuclear size . . . 20
4.4.1 Determining the concentration of TF molecules in the nucleus . . . . 20
4.4.2 Determining the concentration of chromatin binding sites in the nucleus . . . 20
4.4.3 The bound fraction $Q$ depends on the nuclear size . . . 21
4.5 The relative change in apparent number of chromatin binding sites does not depend on TF affinities . . . 21

**5 Supplementary References** **22**

## 1 Supplementary Figures

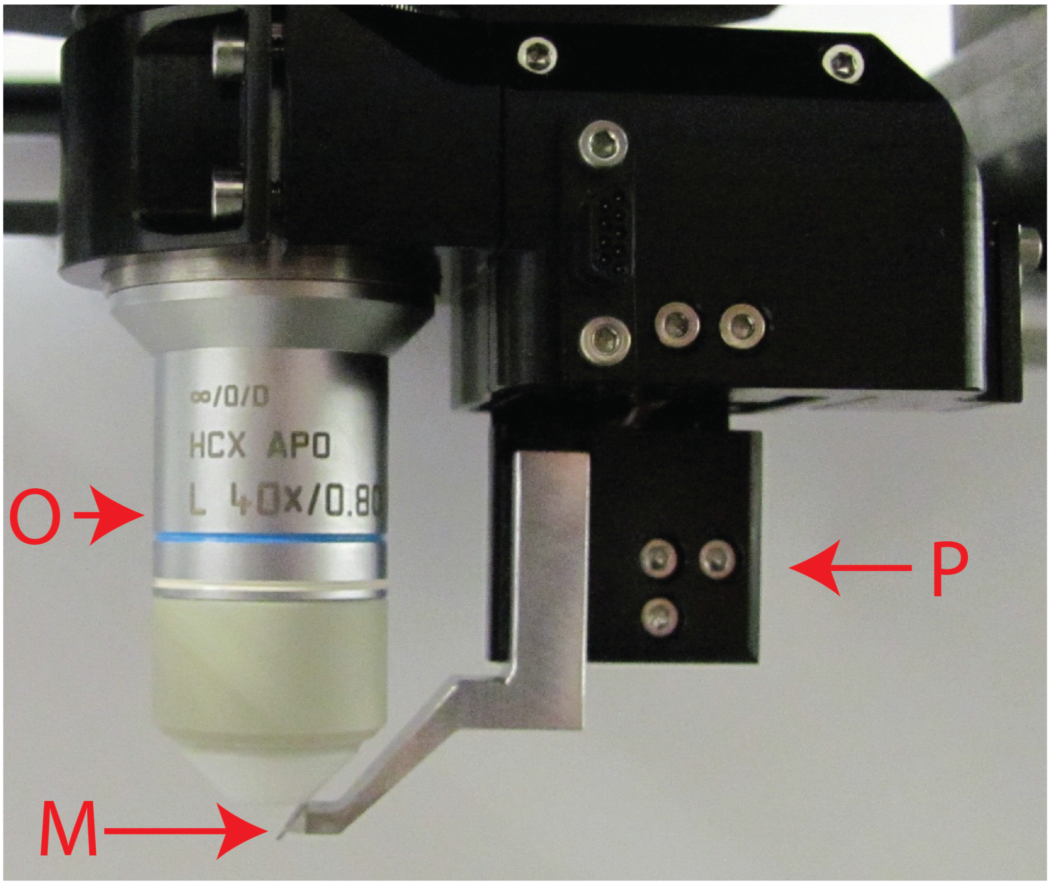


**Supplementary Figure 1**
**Illumination unit of the RLS-microscope.** **O** water dipping objective, **M** micro-mirror, **P** x-y-z micro positioning stage

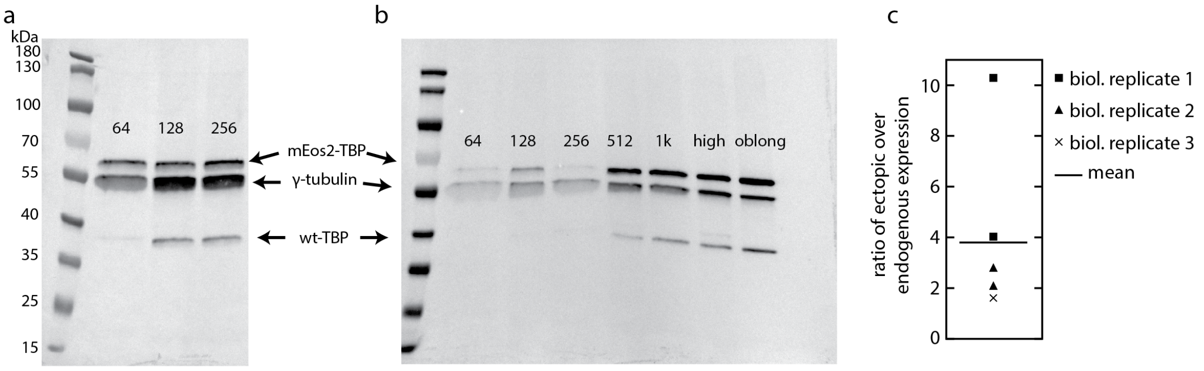


**Supplementary Figure 2**

**Expression of TBP and mEos2-TBP in developing zebrafish embryo.** Expression in embryos with (**a**) 64, 128 and 256 cells, 15.2 pg of total protein loaded and (**b**) 64, 128, 256, 512, 1000 cells, high and oblong stage, 7.6 pg of total protein loaded. Total protein concentration in the embryo lysate loaded was determined by BCA assay. (**c**) Quantification of overexpression level in 1000 cell embryos. Data is from three biological replicates (square, triangle and cross), of which two were replicated technically. The average (horizontal line) is calculated from the pre-averaged technical replicates.

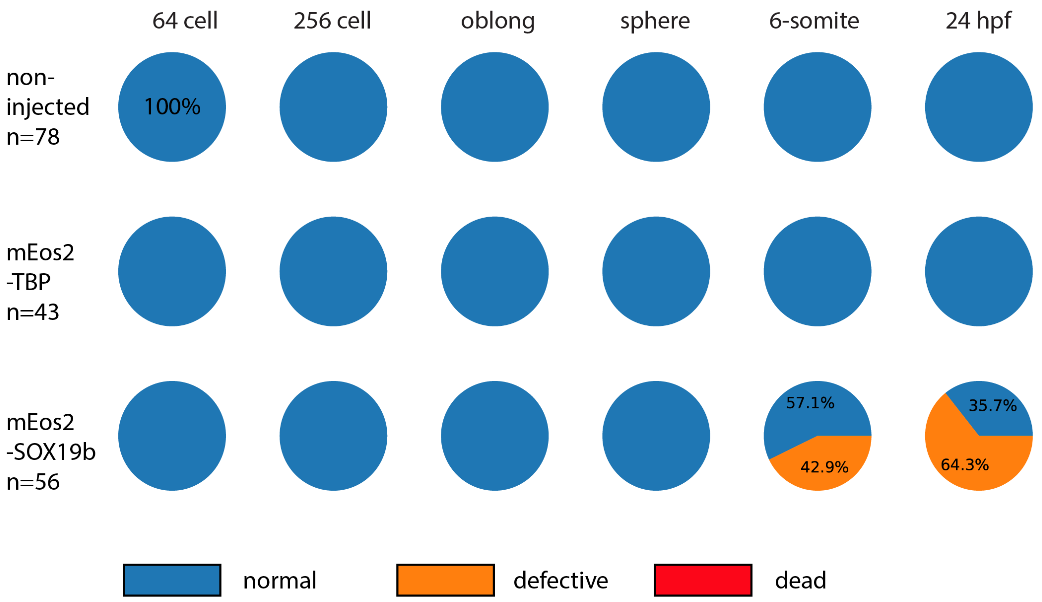


**Supplementary Figure 3**

**Quantification of phenotype occurrence** Occurrence of wild-type (blue), defective (orange) or dead (red) phenotypes in non-injected embryos (n=78 embryos) and upon injection of mEos2-TBP (n=43 embryos) or mEos2-Sox19b (n=56 thereof 24 defective at 6-somite stage and 36 defective after 24 hours) together with eGFP-Lap2$\beta$ at 25°C. 24 hours after fertilization non-injected embryos and mEos2-TBP injected embryos reached prim-5 stage, while mEos2-Sox19b injected embryos were mainly defective. None of the embryos died during the course of the experiment.

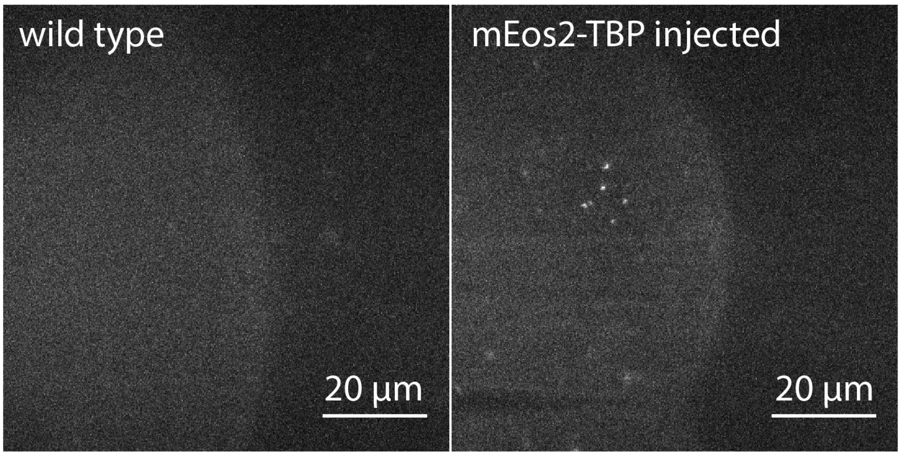


**Supplementary Figure 4**
**Comparison of wild type and mEos2-TBP injected embryos.** Wild type embryo and mEos2-TBP injected embryos under illumination with 561 nm laser light after photoactivation with 405 nm laser light. The wild type embryo does not show fluorescent spots. For both embryos at 128-cell stage exposure time was set to 50ms.

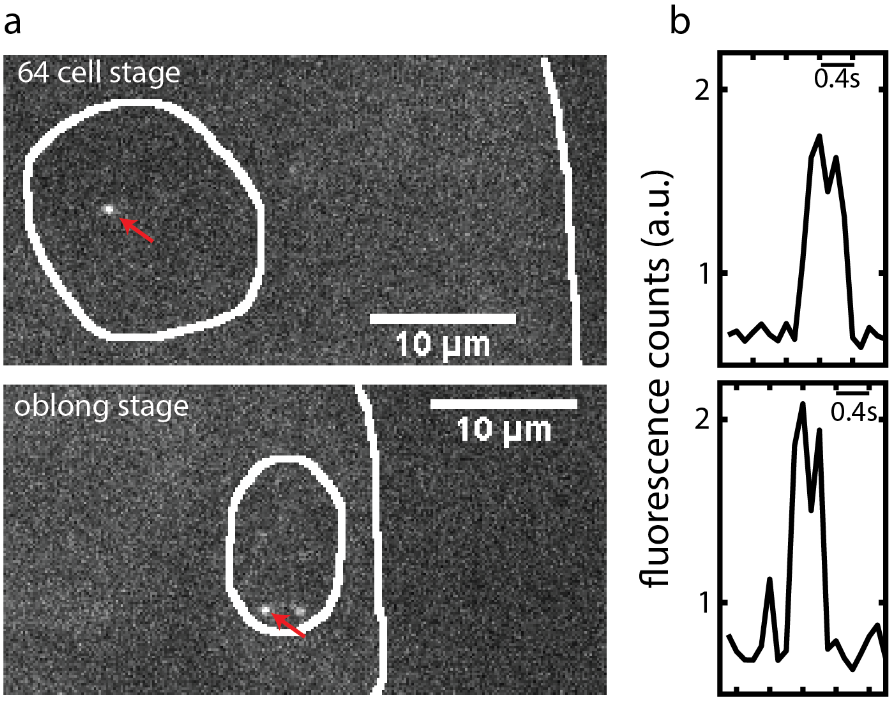


**Supplementary Figure 5**
**Examples of single mEos2-Sox19b molecules.** (**a**) fluorescence images of mEos2-Sox19b in a nucleus of a 64-cell stage embryo and an oblong stage embryo. The surface of the animal cap and the outline of the nucleus are indicated (white lines). Red arrows point to single mEos2-Sox19b molecules. (**b**) Time traces of mEos2-fluorescence of the molecules indicated in the left panels.

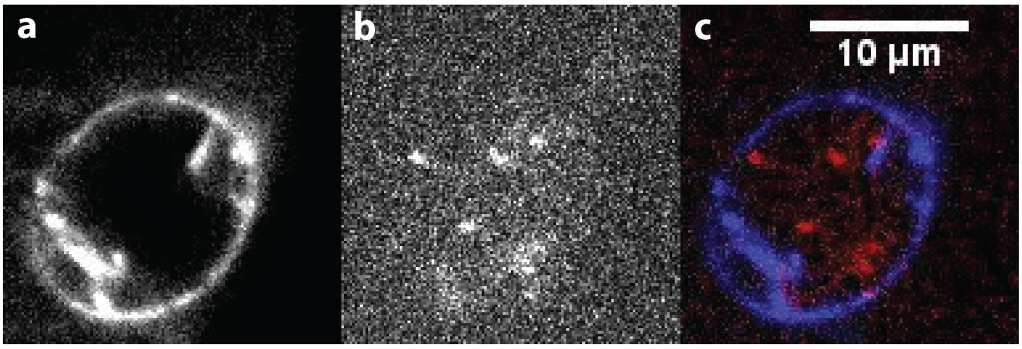


**Supplementary Figure 6**
**Visualization of mEos2-TBP in the nucleus.** (**a**) To localize the nucleus inside the cell, eGFP-Lap2$\beta$ was excited with 488 nm laser light and fluorescence was detected on an EMCCD camera with 50 ms integration time. (**b**) To visualize the TBP molecules inside the cell, mEos2-TBP was excited with 561 nm laser light after photoactivation with 405 nm laser light and fluorescence was detected on an EMCCD camera with 50 ms integration time. (**c**) Merged images A+B in false-colors with separately adjusted contrast for better visibility.

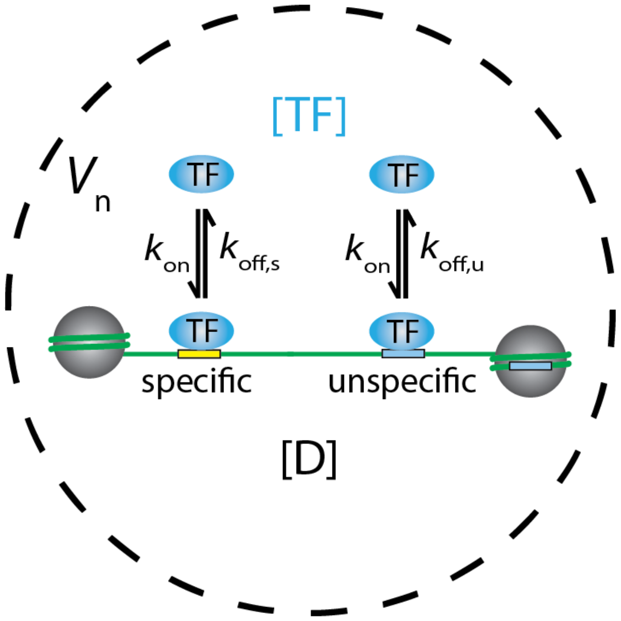


**Supplementary Figure 7**
**Kinetic scheme of the concentration model.** Following the law of mass action, the concentration of TF-chromatin complexes depends on the concentration of TF, [TF], the concentration of chromatin binding sites, [D], the on-rate $k_{on}$ as well as the off-rate $k_{off,u}$ from unspecific sequences and $k_{off,s}$ from specific target sites. Concentration of TF and chromatin is influenced by the size of the nucleus $V_n$.

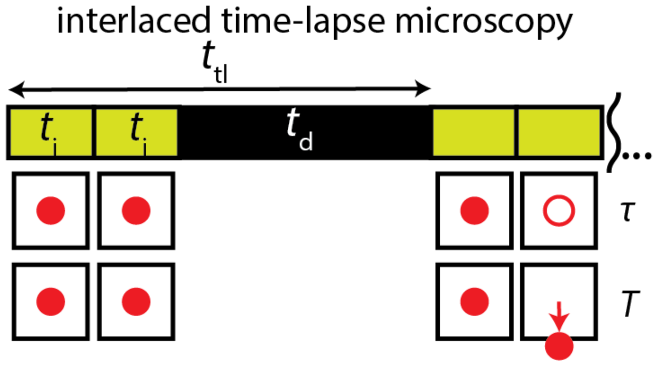


**Supplementary Figure 8**
**Illumination scheme in ITM.** Two successive illumination times $t_i$ are followed by a dark time $t_d$ over a total duration $t_{tl}$. A fluorescent spot trajectory (red dot) identified as a bound molecule can be terminated by two events: by photo-bleaching after time $\tau$ (empty red circle) or unbinding of the molecule after time $T$ (red filled dot leaving the specified area)

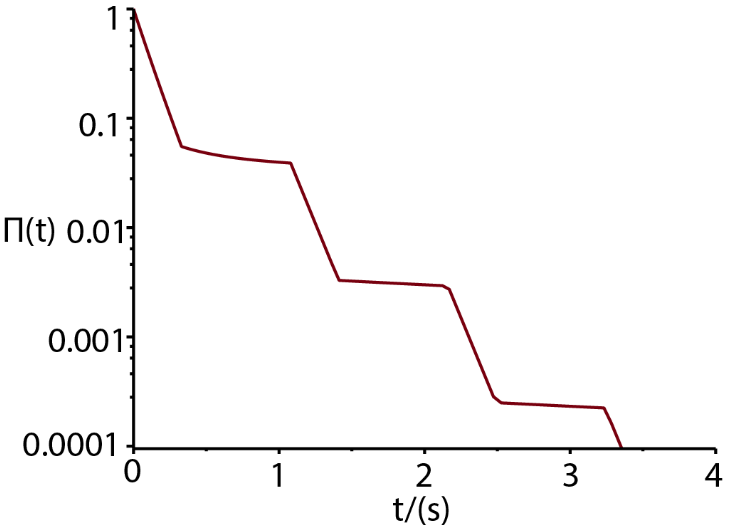


**Supplementary Figure 9** Survival probability of the fluorescent on-state over time in interlaced time-lapse microscopy

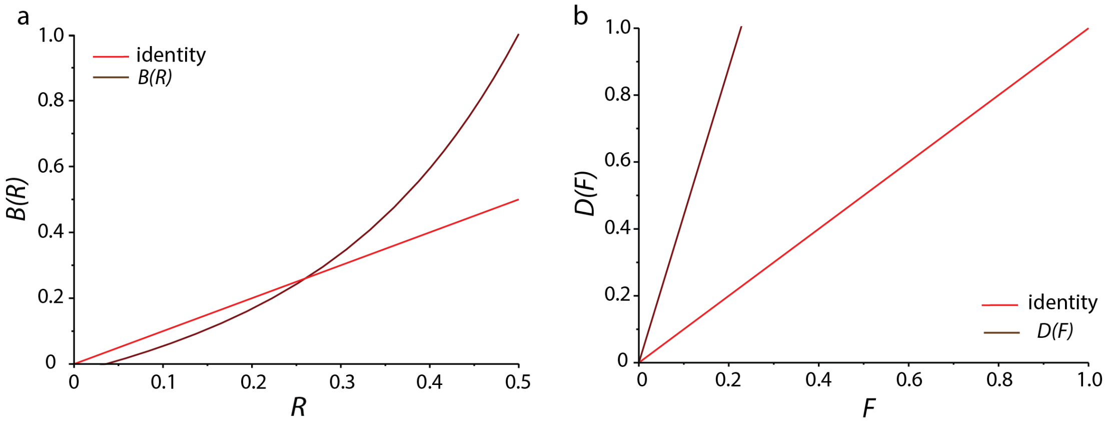


**Supplementary Figure 10**

**Correction of parameters extracted by ITM.** Numbers for stable or short bound molecules obtained by ITM need correction, as photobleaching of the mEos2 fluorophore and unbinding of stable bound TBP molecules before the termination of a dark time biases the distribution towards short binding or non-binding events. (**a**) The stable bound proportion of all chromatin-bound molecules molecules, $B$, can be under- or overestimated by the ratio $R$ of molecules surviving at least one dark time to all molecules surviving at least two frames, that is experimentally determined by ITM and needs to be corrected. As an example, values of the rate constants for long ($k_1$) and short ($k_2$) binding and bleaching ($k_b$) of mEos2-TBP extracted by time-lapse microscopy are $k_1 = 0.15\frac{1}{s}$, $k_2 = 3.92\frac{1}{s}$, $k_b = 7.30\frac{1}{s}$. The time settings for the illumination time ($t_i$) and the total time-lapse duration $t_{tl}$ are $t_i = 0.165s$, $t_{tl} = 1.08s$. Evaluating and inverting equation 13 for these values using the computer algebra program MAPLE 17 and plotting $B$ as a function of $R$ is shown. In red the ideal one-to-one correction is shown. Below a ratio of $R$ of approx. 32%, $B$ is overestimated. Above a ratio of $R$ of approx. 32% $B$ is underestimated. (**b**) The percentage of all bound molecules compared to all molecules present in the nucleus, $D$, can be addressed by ITM. According to Supplementary Methods section 3.3 we can give a correction for the ratio $F$ of all molecules surviving at least two frames compared to all detected molecules. Here as well, molecules bleaching within the first frame will falsely be counted as non-bound molecules. As an example, we plot here the correction curve that is obtained using equation 17 and the values $k_1 = 0.15\frac{1}{s}$, $k_2 = 3.92\frac{1}{s}$, $k_b = 7.30\frac{1}{s}$ and a exemplary value of 0.3 for $B$.

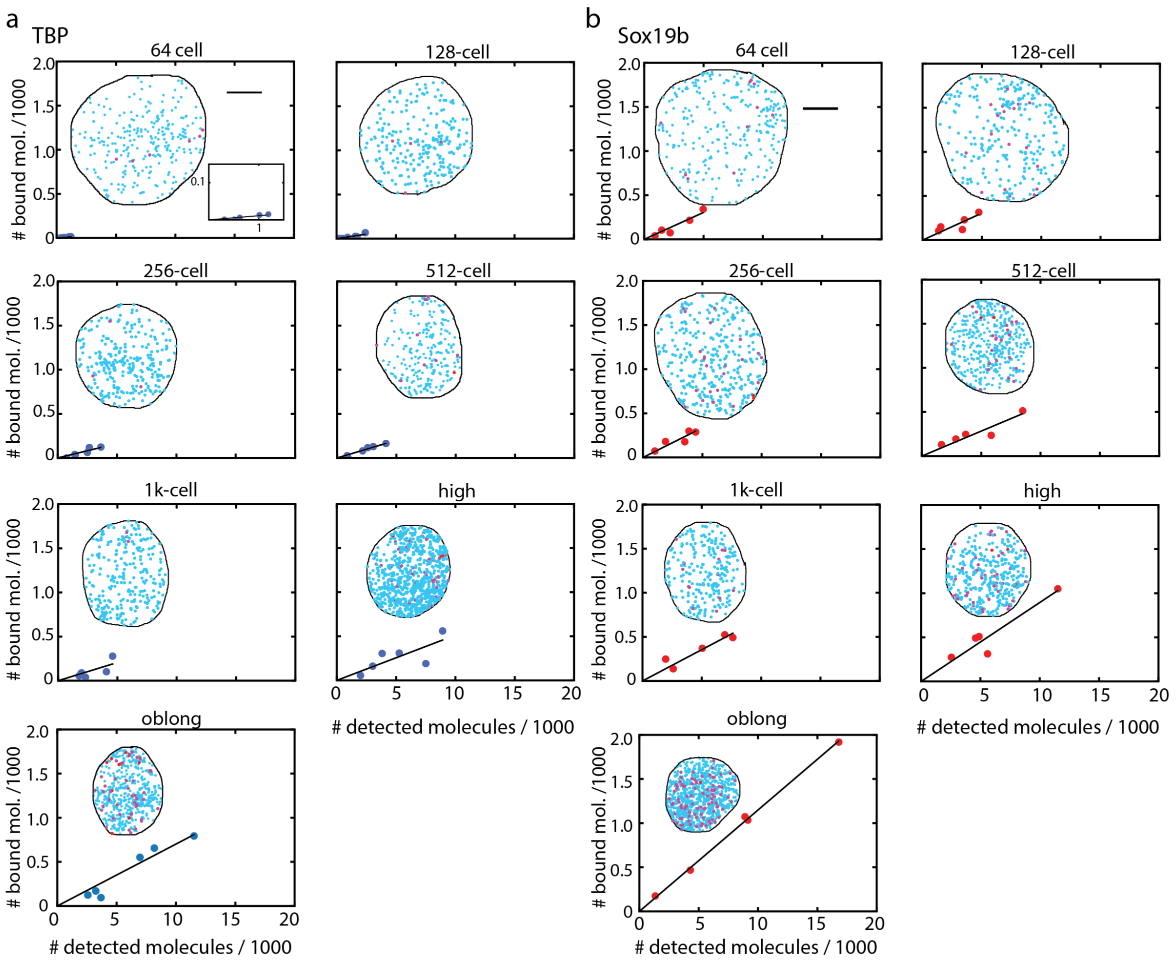


**Supplementary Figure 11.**

**Relation between bound molecules and all detected molecules.** Every dot represents molecules classified as bound compared to all detected molecules for one embryo injected with (**a**) mEos2-TBP and (**b**) mEos2-Sox19b. For every developmental stage the number of bound molecules varies linearly with the number of all detected molecules, with different coefficients of proportionality this holds for TBP and Sox19b. The figure includes in total 120572 molecules from 5 embryos (64-cell stage) and 6 embryos (later stages) for TBP and 158912 molecules from 5 embryos for Sox19b. *Insets* time-projection of all molecules within a movie classified as unbound (blue), short bound (magenta) and long bound (red). Scale bars are 5 $\mu m$.

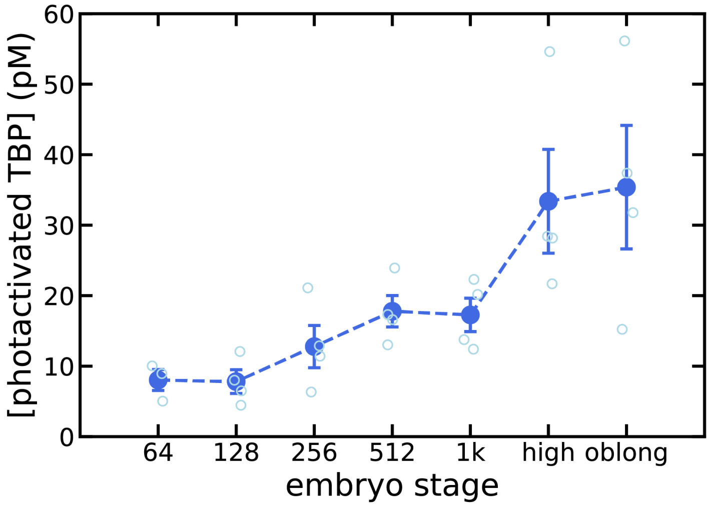


**Supplementary Figure 12.**
**Nuclear concentration of photoactivated mEos2-TBP.** Counting all detected fluorescent molecules within a light-sheet of known thickness yields absolute concentrations of photocativated, fluorescent mEos2-TBP molecules. The figure includes in total 2445 molecules in 3 embryos (64-cell stage) and 4 embryos (later stages). Data are represented as mean $\pm$ s.e.m..

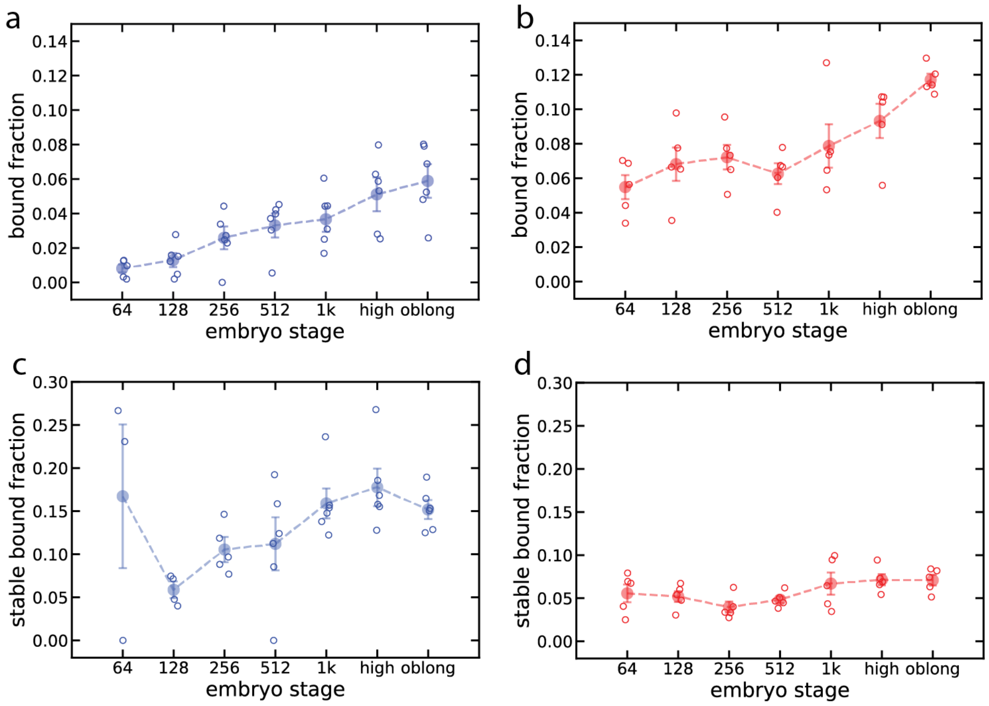


**Supplementary Figure 13.**

**Raw ratios of molecules sorted into binding time classes.** (**a** and **b**) Ratio of bound to all detected molecules for (a) TBP and (b) Sox19b before correction. Open circles display the ratios observed for single embryos in a certain stage. Closed circles represent mean ± s.e.m. Panels include 120572 molecules from 5 embryos (64-cell stage) and 6 embryos (later stages) for TBP and 158912 molecules from 5 embryos for Sox19b (**c** and **d**) Ratio of stable bound to all detected molecules for (c) TBP and (d) Sox19b before correction. Open circles display the ratios observed for single embryos in a certain stage. Closed circles represent mean ± s.e.m. Panels include in total 5677 molecules from 3 embryos (64-cell stage), 4 embryos (128-cell stage), 5 embryos (256-cell stage) and 6 embryos (later stages) for TBP and 13103 molecules from 5 embryos for Sox19b

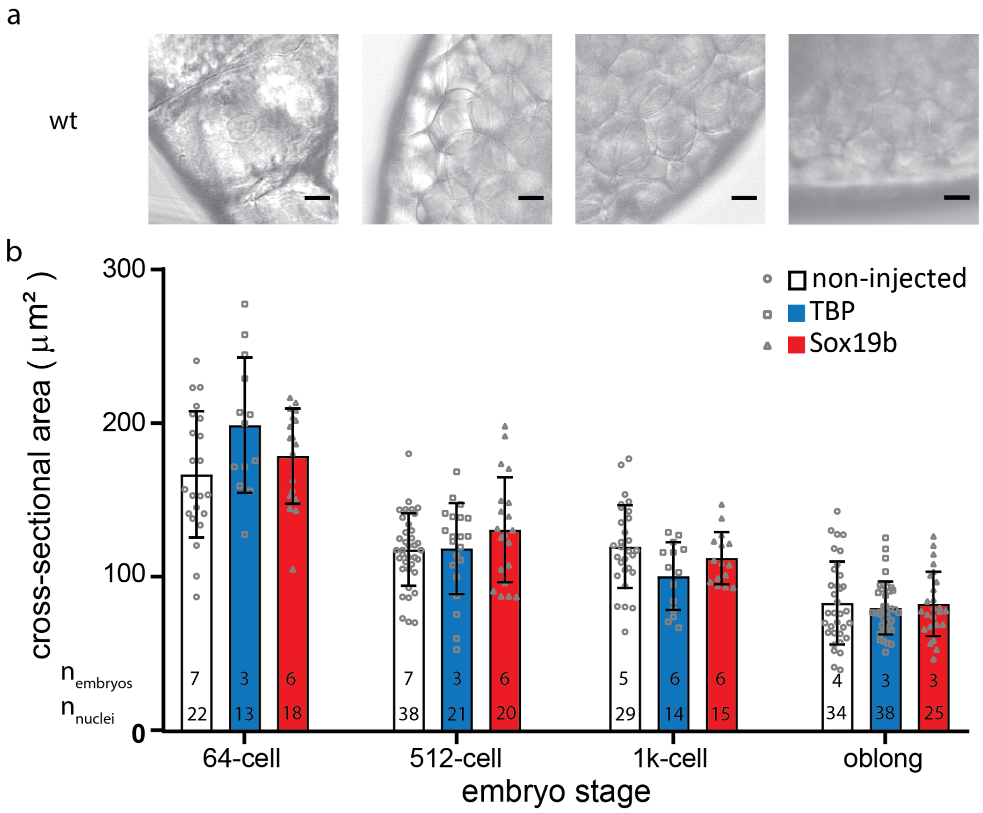


**Supplementary Figure 14.**
**mRNA injection does not alter nuclear size.** The cross-sectional area of a nucleus was identified in bright-field microscopy using a 10x objective (64-cell to 1k-cell stages) or a 40x objective (oblong stage). (**a**) Exemplary images of wt embryos. Scale bar is 15 $\mu$m. (**b**) Comparison of nuclear size of wild type embryos (green) and embryos injected at the 1-cell stage with mRNA encoding for eGFP-Lap2$\beta$ and mEos2-TBP (blue) or respective mEos2-Sox19b (red) at different developmental stages. Data are represented as mean $\pm$ s.d.

# 2 Supplementary Tables

Table 1: Primers used for cloning of fusion proteins

| | |
|---|---|
| Sox19b_BspEI_fwd | ATAATCCGGAatgatgtacagcatgatg |
| Sox19b_XbaI_rev | aaTCTAGATTAgatgtgagtgagggg |
| mEos2_2xHA_BamHI_fwd | GgatcCATGTACCCATACGATGTTCCAGA TTACGCTGGATATCCATATGATGTTCCAG ATTATGCTtcaATGAGTGCGATTAAG |
| mEos2_BspEI_rev | attaTCCGGAGCCAGAACC |

Table 2: Primers used for ChIP-qPCR

| | | |
|---|---|---|
| hmga1a | TAAAGGAGCGGTCGGGACAAA | this work |
| | CTGCTGACAATTCAAACGCAC | |
| brd2a | GACAGCTAGATTGGCCTGTAGA | this work |
| | TATCCGAGTTTCGCGGCCT | |
| apoeb | TAAAGTGAGCAAATGTATGGCC | [1] |
| | TTTGTTGATTAAATCGCTTGTGA | |
| dusp6 | CATATGTTAAGCGGGGTGAAAC | [1] |
| | ATCCTGTCTCCTGTGTCATTTG | |
| pcdh18a | AAGGCCCGTCCCAACTGAGGG | [2] |
| | CTACGTCTCAATCTCCCTGACAGA | |
| genomic control | ATGTTTAGCACTCCAGCGTACTCC | this work |
| | TTTGAGTCCCGAGTCTTGGACGTG | |

Table 3: Antibodies used in this study

| | |
|---|---|
| Western Blot | |
| Mouse-anti-TBP IgG fraction (ab51841) | Abcam |
| Mouse-anti-gamma-tubulin IgG1 (ab11316) | Abcam |
| Goat-anti-mouse IgG alkaline phosphatase (A24527) | Sigma-Aldrich |
| ChIP | |
| Rabbit-anti-HA tag IgG (ab9110) | Abcam |
| Normal Rabbit IgG (# 2729) | Cell Signaling |

Table 4: Complete nucleotide sequence of TBP

| |
|---|
| ATGGAGCAGAACAACAGCCTACCTCCTTTCGCTCAAGGTCTGGCATCTCCACAG |
| GGAGCCATGACGCCTGGCCTGCCCATTTTCAGTCCTATGATGCCGTATGGGACG |
| GGCCTCACACCGCAGCCTGTGCAGAACTCCAACAGCTTGTCCCTCCTGGAGGAG |
| CAACAGAGGCAACAACAGCAGCAGCAGGCGGCCTCACAGCAGCAGGGTGGGATG |
| GTGGGAGGTTCAGGCCAGACGCCCCAGCTTTACCACTCAACACAAGCCGTCTCC |
| ACAACAACAGCTCTGCCAGGCAACACACCACTTTATACCACACCCCTCACCCCT |
| ATGACGCCTATCACTCCTGCCACACCGGCCTCCGAGAGCTCTGGCATCGTCCCG |
| CAGTTACAGAATATTGTGTCCACCGTAAACTTGGGGTGCAAACTTGATTTGAAG |
| ACGATAGCACTTCGAGCCAGAAATGCTGAATATAATCCAAAGCGTTTTGCTGCC |
| GTCATCATGAGAATACGAGAACCCAGAACAACAGCGCTTATCTTCAGCTCGGGG |
| AAGATGGTGTGCACAGGAGCCAAAAGTGAGGAACAGTCTCGATTGGCAGCCAGG |
| AAATATGCCAGAGTGGTGCAGAAGTTGGGTTTCCCTGCCAAATTCTTAGACTTC |
| AAAATCCAGAACATGGTGGGGAGCTGCGATGTCAAGTTTCCCATCCGATTAGAG |
| GGCCTGGTGCTTACCCACCAGCAGTTTAGCAGCTATGAACCGGAGTTATTTCCA |
| GGGTTAATCTACAGAATGATCAAACCCAGAATCGTTCTTTTAATATTTGTTTCA |
| GGCAAAGTCGTACTCACAGGTGCCAAGGTTAGAGGAGAAATCTATGAAGCATTT |
| GAGAATATTTACCCCATCTTAAAAGGATTCAGGAAGACCTCGTAA |

# 3 Supplementary Methods

Consider an ensemble of fluorescent molecules separated into two different classes of binding affinities to chromatin (long and short binding) where the fraction of long or short bound molecules or the binding times undergo a change over time.

## 3.1 Optimized temporal illumination scheme and classification of molecules

To optimize the time needed to collect a sufficient amount of information to sense changes in fraction or binding time, the number of time-lapse conditions is reduced to two. Two frames are recorded without pause and the illumination is interrupted for a certain dark time $t_d$, before two new frames are recorded (see Supplementary Figure 8). Only trajectories of molecules are considered that move less than 165 nm within two adjacent frames. Detected molecules are then separated into trajectories detected only in two frames without a dark time and trajectories surviving at least one dark time. By carefully choosing the dark time, only molecules of the long bound fraction possibly survive the pause time, whereas in the set of trajectories without a dark time molecules of both fractions can be found. By dividing the number of molecules in both classes a concentration-independent measure $R$ for the chromatin affinity of the ensemble can be defined.

$$R = \frac{\text{\# trajectories with dark time}}{\text{\# trajectories with dark time} + \text{\# trajectories w/o dark time}} \tag{1}$$

$R$, however, does not directly reflect the percentage of all specifically bound molecules, since it is biased by i) the portion of specifically bound molecules that bleach before surviving a dark time and ii) the finite probability of a specifically bound molecule to leave its binding site even before having survived a pause. We therefore developed a correction formula to take these two aspects into account.

## 3.2 Linking the ratio stable to all bound molecules to quantitative values

### 3.2.1 Binding time distribution of fluorescent molecules in ITM

If a fluorescent molecule binding to chromatin is observed at time $t = 0$, the signal can be lost due to either bleaching or unbinding from chromatin. Be $T$ a random variable describing the time the molecule stays bound to chromatin and be $\tau$ a random variable describing the time the molecule stays fluorescent then the observed time is given by $\theta = \min(T, \tau)$. If $T$ and $\tau$ are independent the probability for observing a binding time $\theta$ longer than a time $t$ is given by:

$$\Pi(t) = P(\theta > t) = P(\min(T, \tau) > t) = P(\{T > t\} \cap \{\tau > t\}) = P(T > t) \cdot P(\tau > t) \tag{2}$$

For the binding time $T$, the probability density $f_T$ and the survival function $P(T > t)$ for the two-species ensemble with percentage B (stable bound proportion of all bound molecules) and off-rates $k_1$ and $k_2$, is given by:

$$f_T(t) = Bk_1 \exp(-k_1 t) + (1 - B)k_2 \exp(-k_2 t) \tag{3}$$

$$P(T > t) = \int_t^\infty f_T(t')\mathrm{d}t' = B \exp(-k_1 t) + (1 - B) \exp(-k_2 t) \tag{4}$$

For the fluorescence time $\tau$, the probability of bleaching is not constant in time but zero during the dark time. Therefore the probability density for the fluorescence time $f_\tau$ for a trajectory starting with two illuminated frames of duration $t_\mathrm{i}$ each followed by a dark time of duration $t_\mathrm{d}$ yielding a total cycle time of $t_\mathrm{tl} = 2t_\mathrm{i} + t_\mathrm{d}$ is modeled by the following equation 5:

$$f_\tau(t) = \begin{cases} k_\mathrm{b} \exp\left(-k_\mathrm{b} \left\lfloor \frac{t}{t_\mathrm{tl}} \right\rfloor \cdot 2t_\mathrm{i} - k_\mathrm{b}\left(t - \left\lfloor \frac{t}{t_\mathrm{tl}} \right\rfloor \cdot t_\mathrm{tl}\right)\right) & \left(t - \left\lfloor \frac{t}{t_\mathrm{tl}} \right\rfloor \cdot t_\mathrm{tl}\right) < 2t_\mathrm{i} \\ 0 & \left(t - \left\lfloor \frac{t}{t_\mathrm{tl}} \right\rfloor \cdot t_\mathrm{tl}\right) \geq 2t_\mathrm{i} \end{cases} \tag{5}$$

Accordingly for the survival function $P(\tau > t)$:

$$P(\tau > t) = \begin{cases} \exp\left(-k_\mathrm{b} \left\lfloor \frac{t}{t_\mathrm{tl}} \right\rfloor \cdot 2t_\mathrm{i} - k_\mathrm{b}\left(t - \left\lfloor \frac{t}{t_\mathrm{tl}} \right\rfloor \cdot t_\mathrm{tl}\right)\right) & \left(t - \left\lfloor \frac{t}{t_\mathrm{tl}} \right\rfloor \cdot t_\mathrm{tl}\right) < 2t_\mathrm{i} \\ \exp\left(-k_\mathrm{i} \left\lfloor \frac{t}{t_\mathrm{tl}} \right\rfloor \cdot 2t_\mathrm{i}\right) & \left(t - \left\lfloor \frac{t}{t_\mathrm{tl}} \right\rfloor \cdot t_\mathrm{tl}\right) \geq 2t_\mathrm{i} \end{cases} \tag{6}$$

Supplementary Figure 9 shows the survival function $\Pi(t)$ of the observation time $\theta$ for the experimentally found parameters $k_1 = 0.15/s,\ k_2 = 3.92/s,\ k_b = 7.30/s$ and an exemplary value of $B = 1$ (see Supplementary Figure 10 a).

### 3.2.2 Different starting points of the trajectories

Possible starting points for trajectories are the first or the second frame within the illumination cycle. The fractions of trajectories expected to start at each frame can be calculated by the probability to observe a molecule later than at a certain time point $t_1$, if it has bound at a time point $t_0$. Namely for an exponential model:

$$P(t > t_1|t_0) = P(t > t_1 - t_0) \tag{7}$$

The probability for a trajectory starting in either the first or the second frame can therefore be calculated by the likelihood of a molecule having bound in either the dark time plus 1 frame time, or within one frame. Although the latter case seems to be highly unlikely, it is not. The high photobleaching rate and the high off-rate make it more likely for a molecule to have bound in temporal vicinity to the observation time point, than a long time before.
So two cases have to be distinguished:

- First-frame trajectories: these molecules have time from the end of the previous frame during the dark time plus the half of the illumination cycle to bind. Assuming a constant off-rate, we can give a normalized measure for the probability in terms of $\Pi$.

$$p'_{1f} = \frac{\Pi(t_i) - \Pi(t_{tl})}{\Pi(t_i)} \tag{8}$$

- Second-frame trajectories: Here the molecule has to bind within one frame time $t_f$ in the illumination cycle. The equivalent probability is therefore given by:

$$p'_{2f} = 1 - \Pi(t_f) \tag{9}$$

  Furthermore the survival function $\Pi(t)$ has to be re-normalized in this case as the first illumination cycle is missing. This leads to the survival function $\Pi_1(t)$ that is given by:

$$\Pi_1(t) = P(\theta > t|\theta > t_i) = \frac{P(\theta > t)}{P(\theta > t_i)} = \frac{\Pi(t)}{\Pi(t_i)} \tag{10}$$

As one of both cases must be true, the probability has to be normalized:

$$p_{2f} = \frac{p'_{2f}}{p'_{2f} + p'_{1f}} \tag{11}$$

$$p_{1f} = \frac{p'_{1f}}{p'_{2f}} + p'_{1f} \tag{12}$$

By combining the above equations, we find for the probabilistic weight of all molecules surviving a time $t_p$ containing at least one dark time:

$$P(\theta > t_p) = \frac{p_{1f}\Pi(t_{tl}) + p_{2f}\Pi_1(t_{tl})}{p_{1f}\Pi(t_i) + p_{2f}\Pi_1(t_{tl})} \tag{13}$$

This ratio is a function of all decay rates $k_1, k_2, k_b$ of the process as well as of all time settings $t_i$, $t_{tl}$, $t_f$ and the fraction $B$, as defined in equation 3. By inverting the equation one can find the correction factor for the measured ratio $R$, as defined in equation 1 to extract the true fraction $B$.

### 3.3 Correction for the percentage of all bound molecules

Using ITM, it is also possible to compare the total number of all trajectories showing binding at the same spot for two frames with the total number of localizations in the movie. This yields a measure for the percentage of bound and freely diffusing molecules. In our experiment, the number of free molecules, however, will be underestimated as an integration time of 50ms is not able to detect all freely diffusing molecules. Also, the majority of bound molecules is bleaching or unbinding within the first frame and thus counted as unbound molecules. To correct for this we can calculate the percentage of bleached or unbinding molecules within a first frame by computing the survival probability for the molecules over the first frame :

$$P(\theta > t_{\mathrm{i}}) = p_{1\mathrm{f}}\Pi(t_{\mathrm{i}}) + p_{2\mathrm{f}}\Pi_1(t_{\mathrm{tl}}) \tag{14}$$

Thus the probability for mistakenly being counted as non-bound molecule is given by:

$$P(\theta \leq t) = 1 - p_{1\mathrm{f}}\Pi(t_{\mathrm{i}}) + p_{2\mathrm{f}}\Pi_1(t_{\mathrm{tl}}) \tag{15}$$

Let the ratio F of bound to all molecules be calculated by division by the number of all detected molecules:

$$F = \frac{\text{number of bound molecules}}{\text{number of all detections}} \tag{16}$$

To describe the true fraction $D$ of bound molecules, $F$ has to be corrected as follows:

$$D = F \cdot \frac{1}{P(\theta > t_{\mathrm{i}})} \tag{17}$$

The probability therefore is a function of the off-rates, the bleaching rate and the fraction of long and short bound species obtained in the previously described step. An example for a correction curve is shown in Supplementary Figure 10 b.

# 4 Supplementary Notes

Here, we implement a model that describes the formation of complexes between TFs and specific or unspecific binding sites on chromatin in a cell nucleus. The kinetic scheme is depicted in Supplementary Figure 7.

## 4.1 Mathematical description of TF-DNA complex formation

As discussed in the main text we can describe the situation depicted in Supplementary Figure 7 with a chemical reaction equation of the following form

$$\frac{\mathrm{d}[DT]}{\mathrm{d}t} = k_{\mathrm{on}}[D][T] - k_{\mathrm{off}}[DT] \tag{18}$$

With $[T]$: concentration of transcription factor, $[D]$: concentration of chromatin,$[DT]$ : concentration of the TF-DNA complex, $k_{\mathrm{on}}$: kinetic association rate constant to DNA, $k_{\mathrm{off}}$ kinetic dissociation rate constant from DNA. In steady state equilibrium we have

$$\frac{\mathrm{d}[DT]}{\mathrm{d}t} = k_{\mathrm{on}}[D][T] - k_{\mathrm{off}}[DT] = 0 \Rightarrow k_{\mathrm{on}}[D][T] = k_{\mathrm{off}}[DT] \tag{19}$$

Rewriting then yields the law of mass action including the so-called dissociation constant K:

$$K := \frac{k_{\mathrm{off}}}{k_{\mathrm{on}}} = \frac{[D][T]}{[DT]} \tag{20}$$

## 4.2 Exact solution for TF binding to two types of binding sites

For the mathematical description of the situation in the embryo we utilized the law of mass action from equation 20 and extended it to two binding sites. Be the TF concentration again denoted by $[T]$, the concentration of unspecific chromatin binding sites by $[D_{\mathrm{u}}]$ and the concentration of specific chromatin binding sites by $[D_{\mathrm{s}}]$ The model implicates the following equilibria:

$$\mathrm{D_u} + \mathrm{T} \xrightleftharpoons{K_{\mathrm{u}}} \mathrm{D_uT} \text{ with } K_{\mathrm{u}} = \frac{[D_{\mathrm{u}}][T]}{[D_{\mathrm{u}}T]} \tag{21}$$

$$\mathrm{D_s} + \mathrm{T} \xrightleftharpoons{K_{\mathrm{s}}} \mathrm{D_sT} \text{ with } K_{\mathrm{s}} = \frac{[D_{\mathrm{s}}][T]}{[D_{\mathrm{s}}T]} \tag{22}$$

The total number of each species is given by the sum of free and bound molecules:

$$[T]_0 = [T] + [D_{\mathrm{u}}T] + [D_{\mathrm{s}}T] \tag{23}$$

$$[D_{\mathrm{u}}]_0 = [D_{\mathrm{u}}] + [D_{\mathrm{u}}T],\ [D_{\mathrm{s}}]_0 = [D_{\mathrm{s}}] + [D_{\mathrm{s}}T] \tag{24}$$

The dissociation constant for overall TF binding to any binding site on chromatin can be rewritten as follows :

$$K = \frac{[D][T]}{[DT]} = \frac{([D_{\mathrm{u}}] + [D_{\mathrm{s}}])[T]}{([D_{\mathrm{u}}T] + [D_{\mathrm{s}}T])} = BK_{\mathrm{s}} + (1 - B)K_{\mathrm{u}} \tag{25}$$

With the ratio of concentrations of specifically and unspecifically bound molecules denoted by

$$B = \frac{[D_\mathrm{s}T]}{[D_\mathrm{u}T] + [D_\mathrm{s}T]} \tag{26}$$

Assuming the same on-rate $k_\mathrm{on}$ for both specific and unspecific binding sites we find:

$$K = BK_\mathrm{s} + (1-B)K_\mathrm{u} = \frac{Bk_\mathrm{s} + (1-B)k_\mathrm{u}}{k_\mathrm{on}} \tag{27}$$

In words, this means that the effective dissociation constant is given as the weighted average of the single dissociation constants of long and short bound molecules.

To determine the values of $B$, we measured the ratio R of the number of specifically bound molecules in the observation volume $V_\mathrm{ob}$ normalized by the number of all bound molecules:

$$R = \frac{[D_\mathrm{s}T]V_\mathrm{ob}}{[D_\mathrm{s}T]V_\mathrm{ob} + [D_\mathrm{u}T]V_\mathrm{ob}} = \frac{[D_\mathrm{s}T]}{[D_\mathrm{s}T] + [D_\mathrm{u}T]} = B \tag{28}$$

$R$ thus links the mathematical description of the model to our measurement.

Let furthermore $[D] = [D_\mathrm{u}] + [D_\mathrm{s}]$ denote the effective concentration of chromatin binding sites. Then the overall reaction resembles the form of the law of mass action:

$$K = \frac{[D][T]}{[DT]} \tag{29}$$

Hence, we can utilize the simplified equation 29 for the mathematical operations in the following paragraphs.

## 4.3 The bound fraction of TFs

As shown in Figure 4, Supplementary Figure 8 and pointed out in the main text, we do not observe saturation effects. We therefore can assume that the concentration of chromatin binding sites does not change significantly due to binding of TF

$$[D] = [D_0] - [DT] \approx [D_0] \tag{30}$$

Rewriting the law of mass action with this approximation yields a measure of the bound fraction $Q$ of molecules

$$Q := \frac{\text{conc. of bound TFs}}{\text{conc. of all TFs}} = \frac{[DT]}{[DT] + [T]} = \left(1 + \frac{[T]}{[DT]}\right)^{-1} = \left(1 + \frac{K}{[D_0]}\right)^{-1} \tag{31}$$

As it can be seen from the right hand side of the equation above, $Q$ is independent of the concentration of TF.

The definition of the concentration of binding sites as the quotient of the number of binding sites $D_0$ and the nuclear volume $V_\mathrm{n}$ gives $[D_0] = \frac{D_0}{V_\mathrm{n}}$. Therefore we can rewrite equation 31 as

$$Q = \frac{D_0}{D_0 + V_\mathrm{n}\frac{k_\mathrm{off}}{k_\mathrm{on}}} \tag{32}$$

An alternative way to determine the quantity Q is to compare the times spent bound ($t_b$) and freely diffusing ($t_f$) for a single molecule. In an ergodic system the percentage

Q' of time spent in the bound state should equal the percentage of molecules bound, i.e. the bound fraction $Q$.

From the chemical reaction Equation 18 for the times spent in one state by a single molecule we find:

$$t_\mathrm{b} = k_\mathrm{off}^{-1} \text{ and } t_\mathrm{f} = (k_\mathrm{on}[D])^{-1} \tag{33}$$

We assume again non-occurrence of saturation so that $[D] \approx [D_0]$. The portion of time spent in the bound state $Q'$ therefore is given by

$$Q' = \frac{\text{time bound}}{\text{total time}} = \frac{t_\mathrm{b}}{t_\mathrm{b} + t_\mathrm{f}} = \frac{k_\mathrm{off}^{-1}}{k_\mathrm{off}^{-1} + (k_\mathrm{on}[D_0])^{-1}} = \frac{k_\mathrm{on}[D_0]}{k_\mathrm{on}[D_0] + k_\mathrm{off}} \tag{34}$$

The expression at the right hand side above is frequently used as expression for the bound fraction. It can be rearranged to see that it is equivalent to Equation 32 :

$$Q' = \frac{k_\mathrm{on}[D_0]}{k_\mathrm{off} + k_\mathrm{on}[D_0]} = \frac{[D_0]}{[D_0] + \frac{k_\mathrm{off}}{k_\mathrm{on}}} = \frac{D_0}{D_0 + V_\mathrm{n}\frac{k_\mathrm{off}}{k_\mathrm{on}}} = Q \tag{35}$$

Thus, we indeed find that $Q' = Q$ and $Q'$ is independent of the concentration of TF.

To determine the values of Q, we measured the number of all bound molecules in the observation volume $V_\mathrm{ob}$ normalized by the number of all detected molecules, $B'$:

$$B' = \frac{[DT]V_\mathrm{ob}}{[DT]V_\mathrm{ob} + [T]V_\mathrm{ob}} = \frac{[DT]}{[DT] + [T]} = Q \tag{36}$$

$B'$ thus links the mathematical description of the model to our measurement.

## 4.4 The bound fraction depends on the nuclear size

### 4.4.1 Determining the concentration of TF molecules in the nucleus

RLSM intrinsically yields numbers $N^\mathrm{f}$ of fluorescently labeled molecules in the observed volume fraction of the nucleus, thus it is a means to determine actual concentrations.

Be $A_i^\mathrm{s}$ the mean cross-section area of the nuclei in the $i$-th cell cycle, $w$ the width of the light sheet, $N_\mathrm{A}$ Avogadro's number and $N_i^\mathrm{f}$ the number of detected molecules in the $i$-th cycle. We found $w = 3.29\mu m$. Thus for the nuclear concentration of detected molecules in the $i$-th cycle $[T]_i$ we find:

$$[T]_i = \frac{N^\mathrm{f}}{A_i^\mathrm{s} \cdot w \cdot N_\mathrm{A}} \tag{37}$$

This means that given a constant sheet geometry the number of detected molecules per unit area is proportional to the actual concentration of the molecules. By calculating relative values of concentrations the constant parameters $N_\mathrm{A}$ and $w$ are canceled out.

### 4.4.2 Determining the concentration of chromatin binding sites in the nucleus

Assuming that chromatin is unable to leave the nucleus the concentration of chromatin is controlled by the nuclear volume. To determine the nuclear volume in each stage, the cross-section $A_i$ was measured in the $i$-th stage and a spherical shape was assumed.

The volume of the nucleus $V_i^\mathrm{n}$ for the $i$-th stage is then given by:

$$V_i^{\mathrm{n}} = \sqrt{\frac{A_i^3}{36\pi}} \tag{38}$$

Given a certain number of chromatin binding sites $N_i$ in the $i$-th stage and Avogadro's number $N_{\mathrm{A}}$ the molar concentration can be calculated as:

$$[D_0]_i = \frac{N_i}{N_{\mathrm{A}} \cdot V_i^{\mathrm{n}}} \tag{39}$$

The chromatin binding site concentration therefore is highly governed by the volume of the nucleus.

### 4.4.3 The bound fraction *Q* depends on the nuclear size

Combining the above information, we find for the ratio $Q_i$ in the i-th stage

$$Q_i := \left(1 + \frac{K_i}{[D_0]_i}\right)^{-1} = \left(1 + \frac{(R_i k_{\mathrm{off,s}} + (1 - R_i)k_{\mathrm{off,u}}) \cdot N_{\mathrm{A}} \cdot V_i^{\mathrm{n}}}{k_{\mathrm{on}} \cdot N_i}\right)^{-1} \tag{40}$$

In eq. 40 every entity is measured during our RLSM experiment except $D_i = k_{\mathrm{on}} N_i$ that can be solved for to infer the apparent number of chromatin binding sites on chromatin.

## 4.5 The relative change in apparent number of chromatin binding sites does not depend on TF affinities

In case the dissociation rate constant of endogenous TFs and exogenous mEos2-TFs differ by a constant factor $\xi$, we find for the endogenous protein a bound fraction $Q_i^{\mathrm{e}}$

$$Q_i^{\mathrm{e}} := \left(1 + \frac{\xi K_i}{[D_0]_i}\right)^{-1} = \left(1 + \frac{\xi(R_i k_{\mathrm{off,s}} + (1 - R_i)k_{\mathrm{off,u}}) \cdot N_{\mathrm{A}} \cdot V_i^{\mathrm{n}}}{k_{\mathrm{on}} \cdot N_i}\right)^{-1} \tag{41}$$

In Equation 41 every entity is measured except $D_i^{\mathrm{e}} := \frac{k_{\mathrm{on}} \cdot N_i}{\xi}$ that can be treated as fitting parameter to infer the apparent number of chromatin binding sites on chromatin in the $i$-th stage. We find that the apparent number of chromatin binding sites for the endogenous protein can be comparable to the one measured for the exogenous mEos2-TF, if it is normalized to one developmental stage (e.g. stage 6)

$$\frac{D_i^{\mathrm{e}}}{D_6^{\mathrm{e}}} = \frac{D_i}{D_6} \tag{42}$$

Thus, the relative changes in apparent numbers of chromatin binding sites between stages are a quantity that can be extracted from our measurements of exogenous proteins.

# 5 Supplementary References

1. Joseph, S. R. *et al.* Competition between histone and transcription factor binding regulates the onset of transcription in zebrafish embryos. *eLife*, **6**, e23326 (2017).

2. Peterson, S.M.& Freeman, J. L. A benchmark for chromatin binding measurements in live cells. *JoVE*. **30**, 1470 (2009).
