# Supplementary material for: Single-molecule imaging correlates decreasing nuclear volume with increasing TF-chromatin associations during zebrafish development
Source: Nat Commun. 2018 Dec 6;9:5218. doi: 10.1038/s41467-018-07731-8 (PMC6283880; doi:10.1038/s41467-018-07731-8)
Supplement: Supplementary file 6 — Description of Additional Supplementary Files [file 41467_2018_7731_MOESM6_ESM.docx]

Movie descriptions:

**Title:** Supplementary movie 1: Imaging of mEos2-TBP inside the zebrafish embryo at the 64-cell stage.
**Description:** Video was taken with 50ms integration time at a framerate of 20 fps. The surface of the animal cap and the outline of the nucleus are indicated (white lines).

**Title:** Supplementary movie 2: Imaging of mEos2-TBP in the oblong stage at the periphery of the embryo.
**Description:** Video was taken with 50ms integration time at a framerate of 20 fps. The surface of the animal cap and the outline of the nucleus are indicated (white lines).

**Title:**  Supplementary movie 3: Imaging of mEos2-Sox19b inside the zebrafish embryo at the 64-cell stage.
**Description:**  Video was taken with 50ms integration time at a framerate of 10 fps. The surface of the animal cap and the outline of the nucleus are indicated (white lines).

**Title:**  Supplementary movie 4: Imaging of mEos2-Sox19b in the oblong stage at the periphery of the embryo.
**Description:** Video was taken with 50ms integration time at a framerate of 10 fps. The surface of the animal cap and the outline of the nucleus are indicated (white lines).
